# Left Bundle Branch Area Pacing vs. Biventricular Pacing for Cardiac Resynchronization Therapy: A Meta-Analysis

**DOI:** 10.3389/fcvm.2021.669301

**Published:** 2021-05-24

**Authors:** Jiyi Liu, Fengzhi Sun, Zefeng Wang, Jiao Sun, Xue Jiang, Weilong Zhao, Zhipeng Zhang, Lu Liu, Shulong Zhang

**Affiliations:** ^1^Heart Centre, Affiliated Zhongshan Hospital of Dalian University, Dalian, China; ^2^Department of Cardiovascular Medicine, Beijing Anzhen Hospital, Capital Medical University, Beijing, China; ^3^Department of Neuroelectrophysiology, Affiliated Zhongshan Hospital of Dalian University, Dalian, China; ^4^Cardiovascular Medicine Institute, Beijing Tiantan Hospital, Capital Medical University, Beijing, China

**Keywords:** meta-analysis, heart failure, cardiac resynchronization therapy, biventricular pacing, left bundle branch area pacing

## Abstract

**Background:** Left bundle branch area pacing (LBBAP) is a recently proposed method for conduction system pacing. We performed a meta-analysis of controlled studies to compare the clinical outcome in patients who received LBBAP vs. biventricular pacing (BVP) for cardiac resynchronization therapy (CRT).

**Methods:** PubMed, Embase, and Cochrane's Library databases were searched for relevant controlled studies. A random-effect model incorporating the potential heterogeneity was used to synthesize the results.

**Results:** Four non-randomized controlled studies including 249 patients with heart failure (HF) for CRT were included, and the patients were followed for 6–12 months. Compared with BVP, LBBAP was associated with significantly shortened QRS duration [mean difference (MD): −29.18 ms, 95% confidence interval (CI): −33.55–24.80, *I*^2^ = 0%, *P* < 0.001], improved left ventricular ejection fraction (MD: 6.93%, 95% CI: 4.69–9.17, *I*^2^ = 0%, *P* < 0.001), reduced left ventricular end-diastolic dimension (MD: −2.96 mm, 95% CI: −5.48 to −0.44, *I*^2^ = 0%, *P* = 0.02), and improved New York Heart Association class (MD: −0.54, 95% CI: −0.84 to −0.24, *I*^2^ = 65%, *P* < 0.001). Moreover, patients who received LBBAP were more likely to achieve echocardiographic [odds ratio (OR): 5.04, 95% CI: 2.17–11.69, *I*^2^ = 0%, *P* < 0.001] and clinical (OR: 7.33, 95% CI: 1.62–33.16, *I*^2^ = 0%, *P* = 0.01) CRT responses.

**Conclusion:** Current evidence from non-randomized studies suggests that LBBAP appears to be a promising method for CRT, which is associated with more remarkable improvements of symptoms and cardiac function in HF patients with indication for CRT.

## Introduction

For heart failure (HF) patients with reduced ejection fraction and complete left bundle branch block (LBBB), cardiac resynchronization therapy (CRT) with biventricular pacing (BVP) has been established as an effective therapy that has been associated with improved left ventricular (LV) function and clinical symptoms (1, 2). However, about 30% of patients do not respond to CRT delivered by conventional BVP (3). In addition, the procedure of BVP implantation is complex, and for patients with venous malformations or coronary vein stenosis, implantation of LV pacing leads is sometimes technically difficult (4). Subsequently, physiological pacing approaches have been investigated to achieve CRT, including His-bundle pacing (HBP) and left bundle branch area pacing (LBBAP) (5). Although HBP could achieve physiologic electromechanical synchrony by facilitating conduction through the native His-Purkinje system, HBP is associated with high pacing threshold and risk of abnormal sensing, which limited its use for CRT delivering (6). LBBAP is a newly developed physiological pacing strategy that can effectively achieve narrowed QRS waves and improved LV function in HF patients with indication for CRT (7). In addition, compared with HBP, LBBAP is of lower thresholds, higher R wave amplitude, and easier to perform, which makes it a potential optimal technique to deliver CRT (8). Although primary case series reporting LBBAP delivered CRT showed promising results (9), controlled studies comparing the efficacy and safety of LBBAP vs. BVP in HF patients with indication for CRT are rare (10–13). Moreover, results of these studies were not consistent, probably due to the limited number of HF patients included in each study (10–13). Accordingly, we performed a meta-analysis of controlled studies to compare the influences of CRT delivered by LBBAP vs. BVP on QRS duration (QRSd), LV function, clinical symptoms, and CRT response in these patients.

## Methods

This systematic review and meta-analysis was prepared in accordance with the PRISMA (Preferred Reporting Items for Systematic Reviews and Meta-Analyses) (14) and the Cochrane's Handbook (15) guidelines during the study design, implementation, data analysis, and results reporting processes.

### Database Searching

PubMed, Embase, and Cochrane's Library databases were searched for relevant studies using the terms of (1) “left bundle branch pacing” OR “left bundle branch area pacing” and (2) “biventricular” OR “cardiac resynchronization therapy” OR “CRT”. The search was limited to human studies published in English. The references of the related original and review articles were also screened manually for potential relevant studies. The final literature searching was performed on January 16, 2021.

### Study Selection

Studies were included if they fulfilled the following criteria: (1) published as full-length article in English; (2) designed as randomized or non-randomized controlled studies, without restrictions of the sample size and follow-up duration; (3) including patients with HF who underwent CRT with LBBAP or BVP; and (4) reported at least one of the following outcomes during follow-up, including QRSd, echocardiographic parameters [left ventricular ejection fraction (LVEF) and left ventricular end-diastolic dimension (LVEDD)], New York Heart Association (NYHA) class, echocardiographic or clinical CRT response rates, and the incidence of adverse events including all-cause mortality or HF rehospitalization. Echocardiographic CRT response was defined as an LVEF improvement of at least 5% at follow-up compared with that at baseline, and clinical CRT response was defined as decreasing NYHA functional class for at least one grade at the last follow-up compared with the basal value (16). Reviews, editorials, preclinical studies, and single-arm studies without a BVP control group were excluded. When duplications of the data were found, the results of the most recent publications with longer follow-up durations were included in the meta-analysis.

### Data Extraction and Quality Evaluation

Two independent authors performed the literature search, data extraction, and quality assessment according to the predefined inclusion criteria. Discrepancies were resolved by consensus and discussion with another author. The extracted data included the details regarding study and patient characteristics; LVEF, LVEDD, and QRSd at baseline in patients treated with LBBAP or BVP; and follow-up durations. Quality of randomized controlled studies was evaluated with the Cochrane's Risk of Bias Tool (15). Quality of non-randomized controlled studies was evaluated with the Newcastle–Ottawa Scale (NOS) (17). This scale judges the quality of each non-randomized controlled study regarding three aspects: selection of the study groups, the comparability of the groups, and the ascertainment of the outcome of interest.

### Statistical Analyses

Mean difference (MD) was used as the general measures for the outcomes of continuous variables, whereas odds ratio (OR) was used for the categorized variables. The 95% confidence intervals (CIs) for MD and OR were also calculated. The heterogeneity among the included studies was detected by the Cochrane's *Q*-test (15, 18) and the *I*^2^-test (19). An *I*^2^ > 50% indicated significant heterogeneity. A random-effect model was used to pool the results of the included studies because this model was considered to incorporate the potential heterogeneity of the included studies and could therefore retrieve a more generalized outcome (15). Potential publication bias was assessed by visual inspection of the funnel plot as well as the Egger regression asymmetry test (20). RevMan (version 5.1; Cochrane Collaboration, Oxford, UK) software was used for the meta-analysis and statistics.

## Results

### Searching Results

**Figure 1:**
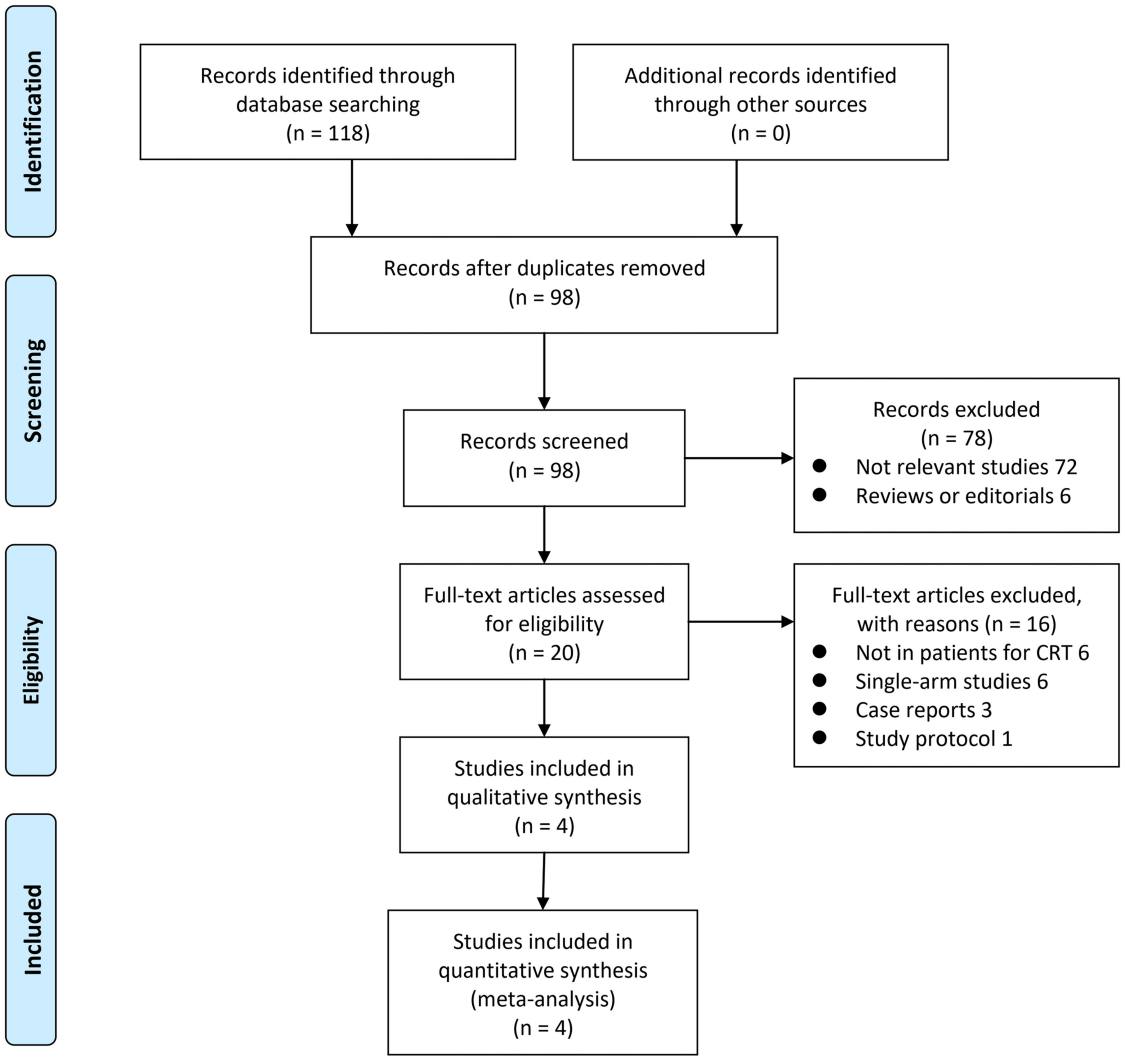
Flowchart of database search and study identification.

The process of literature searching is shown in Figure 1. Briefly, 98 records were retrieved by initial database searching and exclusion of the duplications. By screening *via* title and abstract of the publications, 78 were subsequently excluded, mainly because they were irrelevant to the objective of the current study. The remaining 20 records underwent full-text review, and 16 were further excluded for the reasons listed in Figure 1. Finally, four studies (10–13) were retrieved.

### Study Characteristics and Quality Evaluation

Overall, four prospective non-randomized controlled studies, including 90 HF patients with LBBAP for CRT and 159 patients with BVP for CRT, were included in the meta-analysis (Table 1) (10–13). These studies were all performed in China and published between 2020 and 2021. All of the studies included HF patients with indication for CRT. Patients who received LBBAP and BVP were generally frequency-matched on age; sex; histories of ischemic heart disease; NYHA class; QRSd, LVEDD, and LVEF at baseline; and medications for HF (Table 1). Patients were followed for 6 months in three studies (10–12) and for 12 months in the other one study (13). The quality of the included studies was generally good, with the NOS varied between 8 and 9 points (Table 2).

**Table 1:** Characteristics of the included studies.

**Study**	**Country**	**Design**	**Patients**	**Patient number**		**Mean age (years)**		**Male (%)**		**LVEF (%)**		**LVEDD (mm)**		**QRSd (mm)**		**Follow-up duration (months)**	**Matched variables**
				**LBBAP**	**BVP**	**LBBAP**	**BVP**	**LBBAP**	**BVP**	**LBBAP**	**BVP**	**LBBAP**	**BVP**	**LBBAP**	**BVP**		
Guo et al. (10)	China	NRCT	HF patients for CRT	21	21	66.1	65.1	42.9	42.9	30.0	29.8	64.9	66.7	167.7	163.6	6	Age, sex, histories of IHD, DM, HTN, CKD, AF, intrinsic QRSd, LVEDD, LVEF, NYHA class, and medications for HF
Li et al. (11)	China	NRCT	HF patients for CRT	27	54	57.5	58.5	51.9	61.1	28.8	27.2	66.5	69.4	178.2	180.9	6	Age, sex, histories of IHD, DM, HTN, AF, intrinsic QRSd, LVEDD, LVEF, LAD, NYHA class, and medications for HF
Wang et al. (12)	China	NRCT	HF patients for CRT	10	30	64.8	62.9	90.0	76.7	26.8	26.4	68.6	70.4	183.6	174.6	6	Age, sex, histories of IHD, NYHA class, intrinsic QRSd, LVEDD, LVEF, LAD, and medications for HF
Wu et al. (13)	China	NRCT	HF patients for CRT	32	54	67.2	68.3	43.8	53.7	30.9	30.0	NR	NR	166.2	161.1	12	Age, sex, histories of IHD, DM, HTN, CKD, AF, intrinsic QRSd, MR, LVEF, BNP, NYHA class, and medications for HF

*LVEF, left ventricular ejection fraction; LVEDD, left ventricular end-diastolic dimension; QRSd, QRS-wave duration; NRCT, non-randomized controlled trials; HF, heart failure; CRT, cardiac resynchronization therapy; LBBAP, left branch bundle area pacing; BVP, biventricular pacing; NR, not reported; IHD, ischemic heart disease; DM, diabetes mellitus; HTN, hypertension; CKD, chronic kidney disease; AF, atrial fibrillation; LVESD, left ventricular end-systolic dimension; NYHA, New York Heart Association; LAD, left atrial dimension; MR, mitral regurgitation; BNP, B-type natriuretic peptide*.

**Table 2:** Details of study quality evaluation *via* the Newcastle–Ottawa Scale.

**Study**	**Representativeness of the patient**	**Selection of the controls**	**Ascertainment of intervention**	**Demonstration that outcome of interest was not present at the start of the study**	**Comparability-age and gender**	**Comparability-other factors**	**Assessment of outcome**	**Was follow-up long enough for outcomes to occur**	**Adequacy of follow-up of cohorts**	**Total**
Guo et al. (10)	1	1	1	1	1	1	1	0	1	8
Li et al. (11)	1	1	1	1	1	1	1	0	1	8
Wang et al. (12)	1	1	1	1	1	1	1	0	1	8
Wu et al. (13)	1	1	1	1	1	1	1	1	1	9

### Changes of QRSd, Cardiac Function, and Clinical Symptoms

**Figure 2:**
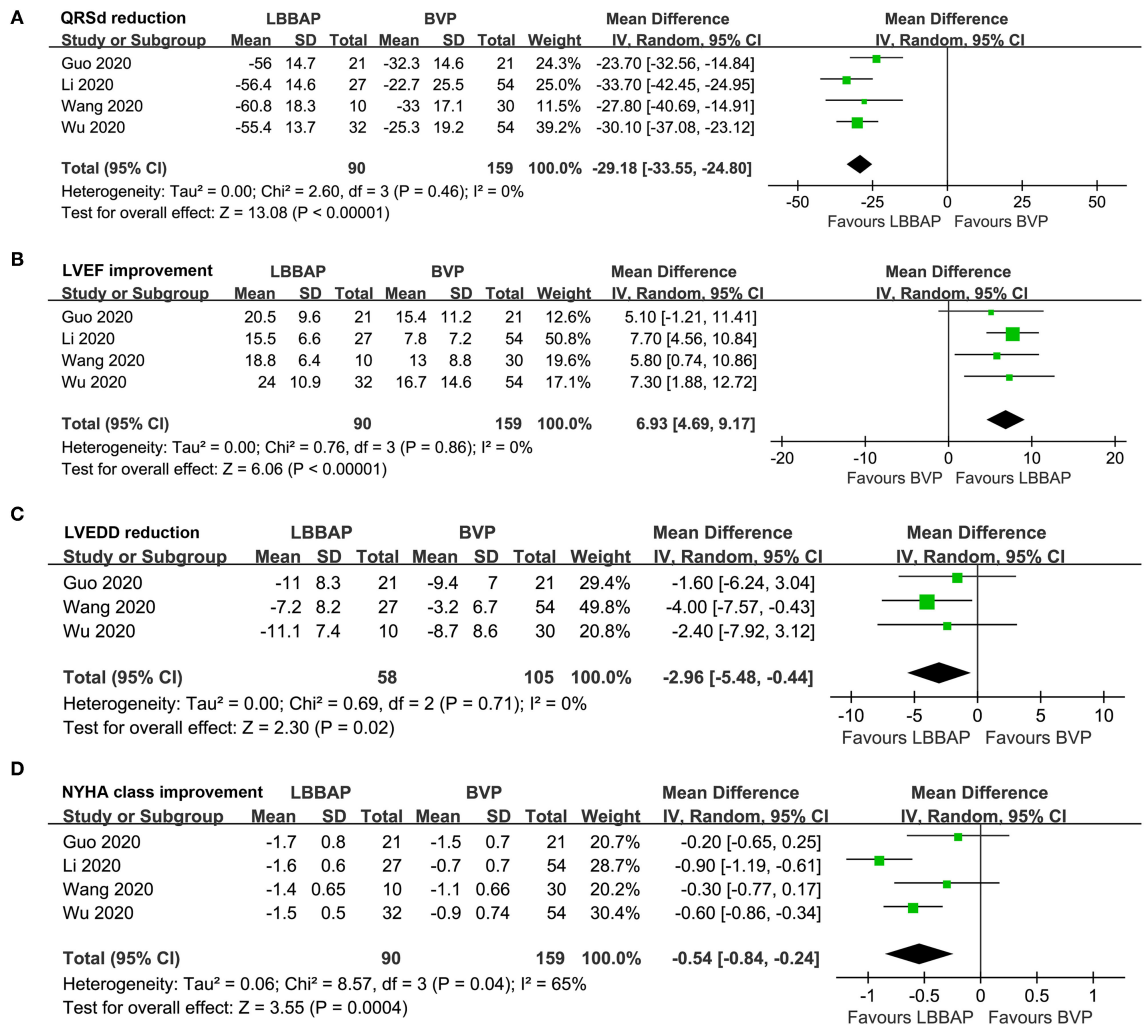
Forest plots for the meta-analysis comparing influences of LBBAP and BVP on QRSd, cardiac function, and clinical symptoms in HF patients with indication for CRT. **(A)** QRSd, **(B)** LVEF, **(C)** LVEDD, and **(D)** NYHA class.

Pooled results with a random-effect model showed that compared with BVP, LBBAP was associated with significantly shortened QRSd (MD: −29.18 ms, 95% CI: −33.55–24.80, *I*^2^ = 0%, *P* < 0.001; Figure 2A), improved LVEF (MD: 6.93%, 95% CI: 4.69–9.17, *I*^2^ = 0%, *P* < 0.001; Figure 2B), reduced LVEDD (MD: −2.96 mm, 95% CI: −5.48 to −0.44, *I*^2^ = 0%, *P* = 0.02; Figure 2C), and improved NYHA class (MD: −0.54, 95% CI: −0.84 to −0.24, *I*^2^ = 65%, *P* < 0.001; Figure 2D).

### CRT Response Rate and Incidence of Adverse Events During Follow-up

**Figure 3:**
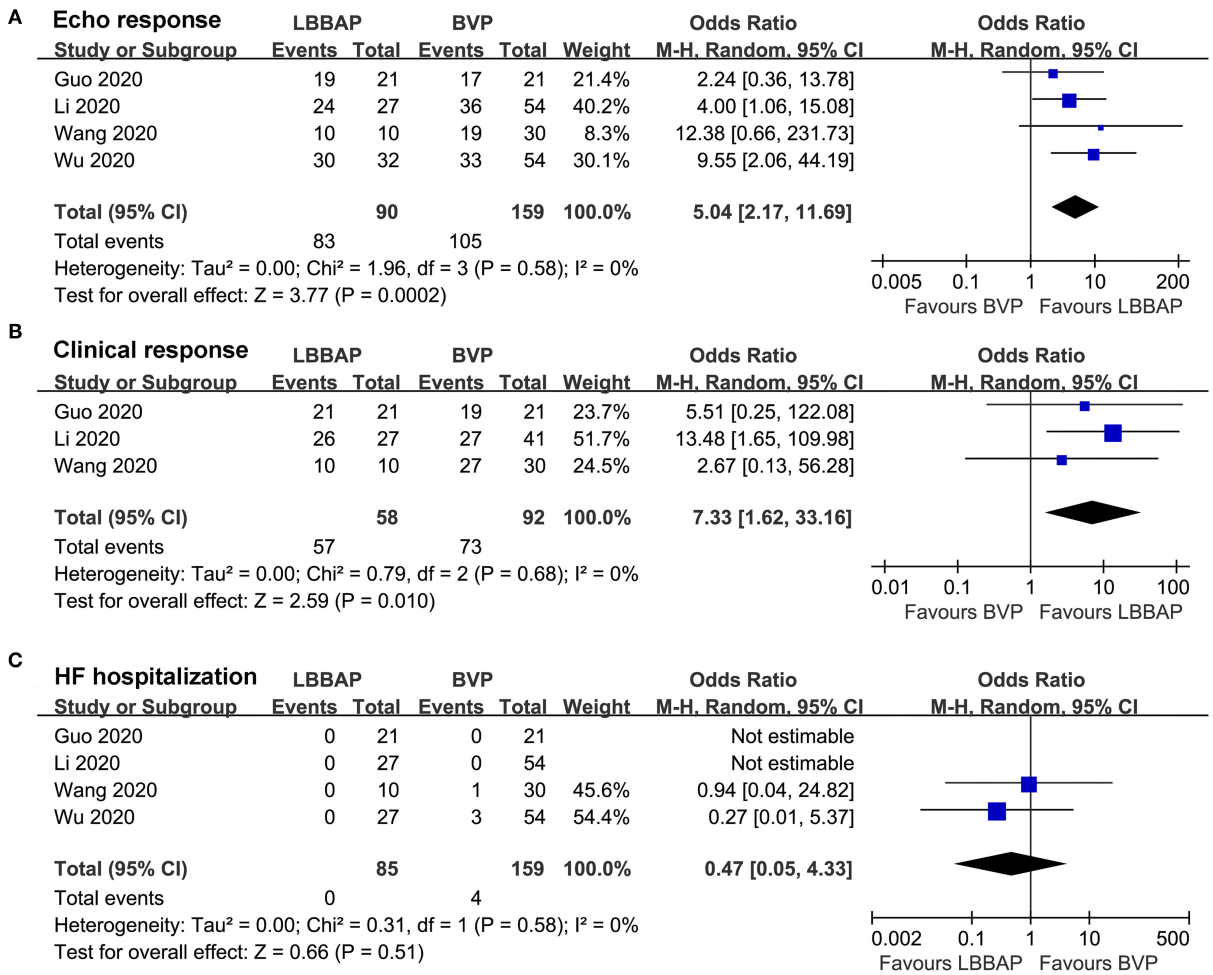
Forest plots for the meta-analysis comparing influences of LBBAP and BVP on CRT response rates and incidence of adverse events during follow-up. **(A)** echocardiographic response, **(B)** clinical response, and **(C)** HF hospitalization.

Pooled results with a random-effect model showed that compared with patients who received BVP, patients who received LBBAP were more likely to achieve echocardiographic (OR: 5.04, 95% CI: 2.17–11.69, *I*^2^ = 0%, *P* < 0.001; Figure 3A) and clinical (OR: 7.33, 95% CI: 1.62–33.16, *I*^2^ = 0%, *P* = 0.01; Figure 3B) CRT responses. No patient died during follow-up, whereas the risk of HF rehospitalization was not statistically different between patients who received LBBAP or BVP (OR: 0.47, 95% CI: 0.05–4.33, *I*^2^ = 0%, *P* = 0.51; Figure 3C).

### Publication Bias

The publication bias for the current meta-analysis was not estimated since only three to four studies were available for each outcome.

## Discussion

In this meta-analysis, by pooling the results of four non-randomized controlled studies, we found that for HF patients with indication for CRT, LBBAP is associated with significantly shortened QRSd, improved LVEF, reduced LVEDD, and decreased NYHA class as compared with conventional BVP at the end of the follow-up. Besides, patients who received LBBAP delivered CRT had higher echocardiographic and clinical response rates than those who received BVP delivered CRT, although the incidence of HF hospitalization was not different between patients from the two groups. These findings suggest that compared with conventional BVP, LBBAP is associated with more remarkable improvements of symptoms and cardiac function in HF patients with indication for CRT, which should be validated in randomized controlled trials (RCTs). Considering the technique feasibility of LBBAP, this novel physiological pacing strategy appears to be promising for HF patients with indication for CRT.

To the best of our knowledge, this study is the first meta-analysis comparing the efficacy between LBBAP and BVP delivered CRT in patients with HF. Since no RCTs regarding the comparative efficacy of LBBAP and BVP delivered CRT have been published, results of the meta-analysis may provide the current evidence-based overview regarding the comparative efficacy of LBBAP and BVP delivered CRT in HF patients during a follow-up of up to 1 year. Previous studies with epicardial activation mapping indicated that electrical dyssynchrony remained despite the use of BVP, suggesting that activation time and pattern could not be corrected to a physiological level by BVP delivered CRT (21). Among new strategies of conduction system pacing, although LBBAP could not achieve normal physiological activation maintained *via* the right bundle as HBP (22, 23), compared with BVP, LBBAP is associated with a significantly further decreased QRSd of −29.2 ms, as evidenced in our meta-analysis. In this meta-analysis, greater improvement of LVEF was achieved by LBBAP delivered CRT compared with BVP delivered CRT, which is paralleled with the more remarkable shortened QRSd in patients after LBBAP delivered CRT. Since a significant association between QRS narrowing and shorter attained QRSd with clinical and echocardiographic CRT responses has been indicated in previous studies, the further shortened QRSd may explain the benefits of LBBAP over BVP on cardiac function and clinical symptoms in HF patients, as well as the increased CRT response during follow-up (24). No significant difference in adverse events, such as HF hospitalization, was observed between groups. However, only four events of HF hospitalization were reported during a follow-up duration of up to 1 year, and our meta-analysis is underpowered for the detection of the potential benefits of LBBAP over BVP on clinical outcomes of HF patients. Large-scale RCTs with longer follow-up durations are warranted.

Although HBP may be more effective to achieve ventricular activation to the physiological level than LBBAP, pilot studies have showed a few technical advantages of LBBAP over HBP, including lower and more stable thresholds, higher implant success rates, and comparable ventricular mechanical synchrony of similar magnitude as HBP (13). With the accumulated experiences and continuous advances in implantation techniques, LBBAP may become an alternative strategy to HBP for CRT delivering with conduction system pacing (25, 26).

Our study has limitations. Firstly, as a meta-analysis of non-randomized controlled studies, although key variables have been frequency-matched, we acknowledged that the potential imbalance of other clinical characteristic of the patients may confound the findings. Ongoing RCTs may validate our findings (27). Secondly, the number of studies and patients is quite limited, whereas the findings of the studies seemed very consistent. In addition, patients were followed for 6–12 months in the studies included in the meta-analysis; the potential long-term benefits of LBBAP over BVP need to be investigated in studies with longer follow-up durations. Besides, as mentioned before, our meta-analysis is not of adequate statistical power to detect the potential benefits of LBBAP over BVP on clinical outcomes of HF patients, and large-scale RCTs with adequate follow-up durations are needed to validate the clinical benefits of LBBAP. Finally, the four included studies were all performed in Chinese centers with early performance of LBBAP. The experiences and skills of the surgeons may affect the comparative efficacy between LBBAP and BVP in HF patients for CRT.

In conclusion, results of this meta-analysis showed that compared with BVP, LBBAP is associated with more remarkable improvements of symptoms and cardiac function in HF patients with indication for CRT. These findings suggested that LBBAP appears to be a more promising method for CRT. The benefits of LBBAP over BVP for HF patients with indication for CRT should be validated in high-quality RCTs.

## Data Availability Statement

The original contributions presented in the study are included in the article/supplementary material, further inquiries can be directed to the corresponding author/s.

## Author Contributions

JL, FS, and SZ designed the study. JL and FS performed the database search, study identification, quality evaluation, data extraction, and drafted the manuscript. ZW, JS, and XJ performed the statistical analyses. All authors interpreted the results, revised, and approved the submission of the manuscript.

## Conflict of Interest

The authors declare that the research was conducted in the absence of any commercial or financial relationships that could be construed as a potential conflict of interest.

## References

[1] Katbeh A Van Camp G Barbato E Galderisi M Trimarco B Bartunek J. Cardiac resynchronization therapy optimization: a comprehensive approach. Cardiology. (2019) 142:116–28. 10.1159/000499192 31117077

[2] Wang Z Wu Y Zhang J. Cardiac resynchronization therapy in heart failure patients: tough road but clear future. Heart Fail Rev. (2021) 26:747. 10.1007/s10741-020-10040-2 33277661

[3] Zhu H Zou T Zhong Y Yang C Ren Y Wang F. Prevention of non-response to cardiac resynchronization therapy: points to remember. Heart Fail Rev. (2020) 25:269–75. 10.1007/s10741-019-09834-w 31352624 PMC7046599

[4] Pothineni NVK Supple GE. Navigating challenging left ventricular lead placements for cardiac resynchronization therapy. J Innov Card Rhythm Manag. (2020) 11:4107–17. 10.19102/icrm.2020.110505 32461816 PMC7244170

[5] Ali N Shin MS Whinnett Z. The emerging role of cardiac conduction system pacing as a treatment for heart failure. Curr Heart Fail Rep. (2020) 17:288–98. 10.1007/s11897-020-00474-y 32857325 PMC7496044

[6] Qi J Jia X Wang Z. His bundle pacing for cardiac resynchronization therapy: a systematic literature review and meta-analysis. J Interv Card Electrophysiol. (2020) 59:463–70. 10.1007/s10840-020-00827-6 32748157

[7] Huang W Su L Wu S Xu L Xiao F Zhou X. A novel pacing strategy with low and stable output: pacing the left bundle branch immediately beyond the conduction block. Can J Cardiol. (2017) 33:1736 e1731–1736 e1733. 10.1016/j.cjca.2017.09.013 29173611

[8] Huang W Wu S Vijayaraman P Su L Chen X Cai B. Cardiac resynchronization therapy in patients with nonischemic cardiomyopathy using left bundle branch pacing. JACC Clin Electrophysiol. (2020) 6:849–58. 10.1016/j.jacep.2020.04.011 32703568

[9] Zhong C Xu W Shi S Zhou X Zhu Z. Left bundle branch pacing for cardiac resynchronization therapy: a systematic literature review and meta-analysis. Pacing Clin Electrophysiol. (2021). 10.1111/pace.14174 33491220

[10] Guo J Li L Xiao G Ye T Huang X Meng F. Remarkable response to cardiac resynchronization therapy via left bundle branch pacing in patients with true left bundle branch block. Clin Cardiol. (2020) 43:1460–8. 10.1002/clc.23462 32960993 PMC7724211

[11] Li X Qiu C Xie R Ma W Wang Z Li H. Left bundle branch area pacing delivery of cardiac resynchronization therapy and comparison with biventricular pacing. ESC Heart Fail. (2020) 7:1711–22. 10.1002/ehf2.12731 32400967 PMC7373885

[12] Wang Y Gu K Qian Z Hou X Chen X Qiu Y. The efficacy of left bundle branch area pacing compared with biventricular pacing in patients with heart failure: a matched case-control study. J Cardiovasc Electrophysiol. (2020) 31:2068–77. 10.1111/jce.14628 32562442

[13] Wu S Su L Vijayaraman P Zheng R Cai M Xu L. Left bundle branch pacing for cardiac resynchronization therapy: nonrandomized on-treatment comparison with his bundle pacing and biventricular pacing. Can J Cardiol. (2021) 37:319–28. 10.1016/j.cjca.2020.04.037 32387225

[14] Moher D Liberati A Tetzlaff J Altman DG. Preferred reporting items for systematic reviews and meta-analyses: the PRISMA statement. BMJ. (2009) 339:b2535. 10.1136/bmj.b2535 19622551 PMC2714657

[15] Higgins J Green S. Cochrane Handbook for Systematic Reviews of Interventions Version 5.1.0. The Cochrane Collaboration (2011). Available online at: www.cochranehandbook.org

[16] Parreira L. Assessing response to cardiac resynchronization therapy: time to settle on some definitive criteria. Rev Port Cardiol. (2018) 37:971–2. 10.1016/j.repc.2018.11.005 30545747

[17] Wells GA Shea B O'connell D Peterson J Welch V Losos M. The Newcastle-Ottawa Scale (NOS) for assessing the quality of nonrandomised studies in meta-analyses (2010). Available online at: http://www.ohri.ca/programs/clinical_epidemiology/oxford.asp

[18] Patsopoulos NA Evangelou E Ioannidis JP. Sensitivity of between-study heterogeneity in meta-analysis: proposed metrics and empirical evaluation. Int J Epidemiol. (2008) 37:1148–57. 10.1093/ije/dyn065 18424475 PMC6281381

[19] Higgins JP Thompson SG. Quantifying heterogeneity in a meta-analysis. Stat Med. (2002) 21:1539–58. 10.1002/sim.1186 12111919

[20] Egger M Davey Smith G Schneider M Minder C. Bias in meta-analysis detected by a simple, graphical test. BMJ. (1997) 315:629–34. 10.1136/bmj.315.7109.629 9310563 PMC2127453

[21] Ploux S Eschalier R Whinnett ZI Lumens J Derval N Sacher F. Electrical dyssynchrony induced by biventricular pacing: implications for patient selection and therapy improvement. Heart Rhythm. (2015) 12:782–91. 10.1016/j.hrthm.2014.12.031 25546811

[22] Elliott MK Mehta V Sidhu BS Niederer S Rinaldi CA. Electrocardiographic imaging of His bundle, left bundle branch, epicardial, and endocardial left ventricular pacing to achieve cardiac resynchronization therapy. HeartRhythm Case Rep. (2020) 6:460–3. 10.1016/j.hrcr.2020.04.012 32695602 PMC7361176

[23] Herweg B Welter-Frost A Vijayaraman P. The evolution of cardiac resynchronization therapy and an introduction to conduction system pacing: a conceptual review. Europace. (2021) 23:496–510. 10.1093/europace/euaa264 33247913

[24] Bazoukis G Naka KK Alsheikh-Ali A Tse G Letsas KP Korantzopoulos P. Association of QRS narrowing with response to cardiac resynchronization therapy-a systematic review and meta-analysis of observational studies. Heart Fail Rev. (2020) 25:745–56. 10.1007/s10741-019-09839-5 31392534

[25] Su L Wang S Wu S Xu L Huang Z Chen X. Long-term safety and feasibility of left bundle branch pacing in a large single-center study. Circ Arrhythm Electrophysiol. (2021) 14:e009261. 10.1161/CIRCEP.120.009261 33426907

[26] Zhang J Wang Z Zu L Cheng L Su R Wang X. Simplifying physiological left bundle branch area pacing using a new nine-partition method. Can J Cardiol. (2021) 37:329–38. 10.1016/j.cjca.2020.05.011 32428620

[27] Cheng L Zhang J Wang Z Zhou M Liang Z Zhao L. Efficacy and safety of left bundle branch area pacing versus biventricular pacing in heart failure patients with left bundle branch block: study protocol for a randomised controlled trial. BMJ Open. (2020) 10:e036972. 10.1136/bmjopen-2020-036972 32973057 PMC7517551

